# Low-Volume Polyethylene Glycol Improved Patient Attendance in Bowel Preparation Before Colonoscopy: A Meta-Analysis With Trial Sequential Analysis

**DOI:** 10.3389/fmed.2019.00092

**Published:** 2019-05-06

**Authors:** Li-Juan Yi, Xu Tian, Bing Shi, Hui Chen, Xiao-Ling Liu, Yuan-Ping Pi, Wei-Qing Chen

**Affiliations:** ^1^Department of Nursing, Hunan Traditional Chinese Medical College, Zhuzhou, China; ^2^Chongqing Key Laboratory of Translational Research for Cancer Metastasis and Individualized Treatment, Chongqing University Cancer Hospital and Chongqing Cancer Institute and Chongqing Cancer Hospital, Chongqing, China; ^3^Key Laboratory for Biorheological Science and Technology of Ministry of Education (Chongqing University), Chongqing University Cancer Hospital and Chongqing Cancer Institute and Chongqing Cancer Hospital, Chongqing, China

**Keywords:** colonoscopy, polyethylene glycol, bowel preparation, meta-analysis, trial sequential analysis

## Abstract

**Background:** Polyethylene glycol (PEG) has been regarded as the primary recommendation for bowel preparation before colonoscopy. However, a conclusive conclusion has not yet been generated.

**Aim:** We performed this updated meta-analysis to further investigate the comparative efficacy and safety of low volume preparation based on PEG plus ascorbic acid related to 4L PEG.

**Methods:** A systematic search was conducted to retrieve potential randomized controlled trials (RCTs) in PubMed, EMBASE, and Cochrane Central Register of Controlled Trials (CENTRAL) from January 2000 to April 2018. Two independent searchers critically searched all potential citations, extracted data, and appraised risk of bias accordingly. Moreover, we used the STATA 12.0 and trial sequential analysis (TSA) 0.9 to complete all analyses.

**Results:** A total of 13 RCTs enrolling 3,910 patients met inclusion criteria. Meta-analysis based on PP analysis indicated that compared to standard volume PEG regime, low volume regime improved patient compliance RR = 1.01; 95% CIs = 1.00, 1.03; *P* = 0.143 (≥75% intake); RR = 1.07; 95% CIs = 1.00, 1.14; *P* = 0.046 (100% intake), the willingness to repeat the same regime (RR = 1.30; 95% CIs = 1.07, 157; *P* = 0.007), and patient acceptability (RR = 1.18; 95% CIs = 1.07, 1.29; *P* = 0.001), and decreased the overall adverse events (RR = 0.86; 95% CIs = 0.77, 0.96; *P* = 0.009). However, no difference was observed between these two different solutions for bowel preparation efficacy (RR = 0.98; 95% CIs = 0.95, 1.02; *P* = 0.340). These all results were further confirmed by TSA.

**Conclusions:** The effect of low volume regime was not inferior to the standard volume PEG regime, and low volume regime was associated with better compliance when subjects ingested all the solution, willingness to repeat the same regime, higher acceptability, and lower nausea in non-selected population.

## Introduction

Colonoscopy has been deemed to be a critical procedure of early diagnosing lesions in the digestive tract, screening colorectal cancer as well as invasive treatment. But it is worth noting that the efficacy and safety of colonoscopy are mainly related to adequate bowel preparation and patient attendance (1–3). In practice, large volume of preparation solutions is administered to patients who are scheduled to perform colonoscopy. However, it is estimated that ~25 to 33% of patients failed to meet the optimal bowel preparations, the main reason is that the patients are intolerant to volume-related discomfort (4, 5). Published evidences suggested that inadequate bowel preparation is closely associated with lower rates of cecal intubation (6), higher operational difficulty, lower adenoma detection rates and greater financial costs (7–9).

Polyethylene glycol (PEG) remains the principle recommended laxatives for bowel cleansing prior to colonoscopy (10, 11). However, in order to obtain sufficient bowel cleaning, patients will be advised to drink 4 L of fluid, and thus the acceptance and compliance with this given regime will be weakened (12, 13). In addition, these limitations also decreased the courage of patients to be involved in the regular colonoscopy surveillance (14, 15). Therefore, reducing this volume without compromising efficacy is a further challenge. To solve this problem, researchers and practitioners shifted attention to modified products, and several studies have found that low volume PEG combined with ascorbic acid (Asc) may have the potential of improving both patient compliance and the success rate of colonoscopy (16–18). Asc is beneficial because it can enable halving the volume of the lavage solution without loss of efficacy and disgusting taste (19, 20). Several RCTs (21–23) have consistently shown that 2L PEG combined with ASC achieved a similar high degree of cleansing compared with standard volume one. Similarly, the findings from a previous meta-analysis (24) are in accordance with the aforementioned studies. However, this meta-analysis involved a quasi-randomized trial (25) and ignored the variation in adjuvants (Bisacodyl and Simethicone) (26, 27), which potentially damaged the power of summary results.

Considering the above information, we thus undertook this updated meta-analysis to further investigate the efficacy and safety of low volume PEG plus Asc related to traditional volume PEG alone comprehensively for bowel preparation before colonoscopy. We also used trial sequential analysis (TSA) to test whether a conclusive conclusion for a specific outcome can be drawn.

## Methods

We finished this article in line with the Preferred Reporting Items for Systematic Review and Meta-analysis (PRISMA) (28) and the Cochrane Handbook for Systematic Reviews of Interventions (29). The prospective protocol for this systematic review was registered on International Prospective Register of Systematic Reviews (PROSPERO) database, and a unique identifier of CRD42018089827 was approved (30). Moreover, the protocol can be accessed in the journal of ***Medicine*** (31). The written informed consent was not to be needed, because all analyses were completed based on published data.

### Selection Criteria

We pre-specified the inclusion criteria, and studies were considered if the following criteria are met: (1) Population (P): The entire population of adult patients undergoing elective colonoscopy, irrespective of outpatients and inpatients; (2) Intervention (I) and Comparison (C): The trials which investigated the comparative efficacy and safety between 2L PEG combined with Asc and 4L PEG alone were considered, and no other adjuvants was added in both groups; (3) Outcomes (O): Bowel preparation efficacy was regarded as primary outcome, and the secondary outcomes included compliance with recommend regime, willingness to repeat the same regime, acceptability to recommend regime, taste of purgative ingested, and safety; and (4) Study design (S): Only RCTs published in English and Chinese were permitted.

Articles were excluded if it conformed to at least one of the following criteria: (1) essential information which cannot be extracted and obtained from authors; (2) duplicates (derive from the same research group) with poor methodology and insufficient data.

### Definition of Outcomes

The overall quality of bowel preparation was predefined as successful bowel cleansing in our study. For the purposes of the analysis, the successful preparation was reached when conformed to one of following conditions: (a) an Ottawa score of < 5; (b) a Boston Bowel Preparation Scale (BBPS) score of ≥2 for all segments; (c) a grade of either excellent or good on the Aronchik scale; (d) grades A and B according to the Harefield Cleansing Scale; and (e) other non-validated 3-, 4-, or 5-point scales (excellent, good, fair, poor, very poor).

Compliance with the regimen was assessed by asking patients how much dose they have ingested. We predefined good compliance as consumption of ≥75% but < 100% of the regime and excellent compliance as consumption of 100% of the regime. In terms of subjective indexes, willingness to repeat the same regime, acceptability to recommend regime, taste of purgative ingested were measured by using an unofficial questionnaire in each individual study. All adverse events related to bowel preparation were monitored and recorded during colonoscopy. All outcomes introduced above were defined by individual study.

### Identification of Citations

A rigorous electronic search was performed by two independent investigators to collect any potential RCTs investigating the comparative efficacy and safety of two targeted PEG-based regimes in PubMed, EMBASE, and Cochrane Central Register of Controlled Trials (CNETRAL) from January 2000 to April 2018. Search results have been updated weekly in order to timely capture any recent studies, and the latest search was updated on August 30 2018. “colonoscopy,” “polyethylene glycols,” and “random” were used to construct search strings based on medical subject heading (MeSH) and free word which are embedded in specific files involving title, keywords and abstract. All search algorithms were designed for targeted databases, and all search algorithms were documented in Supplementary Data Sheet 2.

In addition, we also replenished the potential studies through manually checked the bibliographies of relevant articles and reviews. Two reviewers independently and critically examined citations by reading the titles, abstracts and full-texts accordingly.

### Data Extraction

A predetermined data extraction table was designed. Whereafter, two reviewers independently extracted the following variables: leading author, publication year, risk of bias, age of participants, sample size, bowel preparation assessment scale, the details of regimes, and outcomes of interest. Besides, we utilized information from the www.clinicaltrials.gov and contacted authors of relevant articles to complete the results of publications when necessary. A third author rechecked all information mutually. Divergences between the two reviewers were resolved by arbitration, and consensus was accomplished after discussion.

### Quality Assessment

Two reviewers independently appraised the quality (29, 32) of all included articles by adopting the modified Cochrane risk of bias assessment tool. Evaluation domains including randomization sequence generation, allocation concealment, blinding of participants, blinding of study personnel, blinding of outcome assessors, incomplete outcome data, selective reporting and other bias were assessed. Besides, these assessment results would be cross-checked. The risk of each domain were rated as “high risk of bias,” “unclear risk of bias” or “low risk of bias” according to the match level between extractive information and evaluation criteria (33). Any conflicting result was resolved by discussing with a third author.

### Statistical Analysis

We used STATA software version 12.0 (Stata Corp., College Station, Texas) to perform statistical analyses. Dichotomous data was expressed as relative risk (RR) and 95% confidence intervals (CIs). Heterogeneity were qualitatively evaluated by Cochrane's Q test, and the proportion of overall variation that is attributable to between-study heterogeneity was quantitatively evaluated by *I*^2^ statistic (34, 35). We analyzed the clinical diversity and methodological comparability of every suitable study firstly according to the characteristics of the participants, research design and method, intervention regimes, and measurement and statistical analysis of outcomes. If the clinical characteristic and methodology are considered heterogeneity, qualitative analysis would be used. If not, we would use the Cochrane's *Q*-test to qualitatively evaluate the heterogeneity in studies in terms of each outcome (36). Moreover, the level of heterogeneity would be also quantified by the *I*^2^ statistic. If *I*^2^ is < 50%, the suitable studies would be considered to be homogeneous; in contrast, the pooled results would be affected by substantial heterogeneity. We adopted random-effect model based on Mantel-Haenszel (M-H) or inverse variance (IV) approach to perform all analyses. As to the compliance with recommend regime, subgroup analyses will be planned according to the total consumption of the regime. If the number of studies analyzed in single outcome is more than 10, we detected potential publication bias by visual inspection for funnel plots asymmetry and the Egger test (37). If study with multiple-arm design is included, we will extract the data from intervention groups which are up to the inclusion criteria according to the recommendations proposed by Cochrane Collaboration (29). In order to keep the results more extract, we adopted the intention-to-treat (ITT) analysis and the per protocol (PP) analysis simultaneously in all interest outcomes which can achieve quantitative analysis. In our study, because we cannot confirm the outcome of all patients who withdrew from included trials, therefore ITT analysis defines such patients in experimental group and control group as treatment failure. Moreover, we also performed a sensitivity analysis to determine the bowel preparation efficacy including only the studies that used only split-dose regimens and those that included only outpatient.

### Trials Sequential Analysis

Random error which is a contributor to false positive or negative results will result from repeated significance test of sparse and accumulated data (38–40). Thus, sequential analysis has been proposed to decrease the risk of type I errors, and modified method (TSA) has also been adopted to analyze the pooled results of meta-analysis (38). The quantification of the required information size (RIS) is a major factor to realize the TSA. In the present study, we calculated the RIS adjusted for diversity because the heterogeneity adjustment with *I*^2^ will underestimate the RIS value. The TSA was performed at the level of an overall 5% risk of a type I error and 20% of the type II error (a statistical test power of 80%) (41). If the Z-curve across the monitoring boundary, then we can draw the conclusion of getting credible conclusion before surpassing the RIS line. If the Z-curve across the futility boundary, then we can come to the conclusion of this intervention have no effect for this outcome even though the RIS was not reached. The reliable conclusion can be drawn if the adjusted monitory boundary was surpassed and/or RIS was reached. We estimated the RIS based on the empirical data autogenerated from software according to the data input (42). TSA software (version 0.9 beta) was available at http://www.ctu.dk/tsa/.

## Results

### Identification and Selection of Trials

The initial literature search yielded 1,329 records, and two citations were identified through manual searching and gray literature searching. Exactly 654 articles were eliminated by using the function of duplicate checking embedded in EndNote software. After screening the title, abstract and full-text of all identified studies, 13 eligible articles including 3,910 participants ultimately met our eligibility criteria.

**Figure 1:**
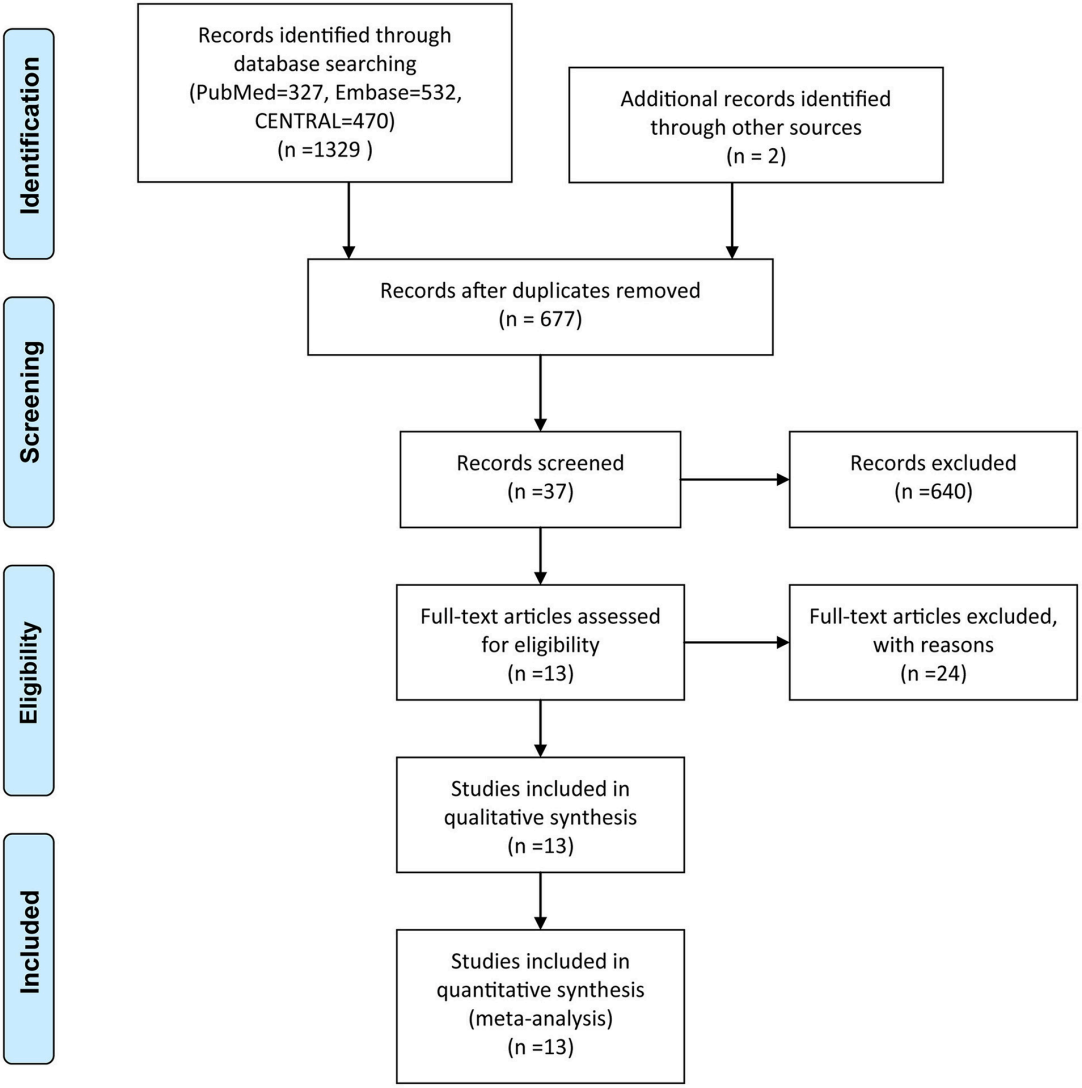
Flow diagram of capturing and selecting searches.

Among these articles, Jung et al. (43) conducted a three-arm trial, we removed a set of data from one group considering the comparability and homogeneity between groups. Besides, one trial with a four-arm study was divided into two RCTs according to the purpose of the study. A flow chart detailing the search strategy and resulting outcome was depicted in Figure 1.

### Characteristics of Included Trials

The characteristics of 13 trials (14 RCTs) (13, 21–23, 43–51) incorporated into this meta-analysis are shown in Table 1. These studies were published between 2007 and 2016. Sample size of each eligible study ranges from 22 to 218.

**Table 1:** The basic characteristics of all eligible studies.

**Study**	**Type of population**	**Sample size**		**Average age**		**Assessment scale**	**Bowel preparation regimes**		**Outcomes**
		**E**	**C**	**E**	**C**		**E**	**C**	
Clark et al. (51)^*^	n.r.	145	149	n.r.	n.r.	n.r.	2L PEG+Asc with split dosage	4L PEG with split dosage	BPE
Lee et al. (50)^*^	n.r.	34	22	NR	n.r.	n.r.	2L PEG+Asc	4L PEG	BPE, WRSP, TPI
Park et al. (47)^*^	n.r.	132	119	n.r.	n.r.	Non-validated 5-point scale	2L PEG+ Asc with split dosage	4L PEG with split dosage	BPE
Ell et al. (22)	Inpatient	153	155	58.0 ± 14.7	59.6 ± 16	Non-validated 5-point scale	2L PEG+Asc with split dosage	4L PEG with split-dosage	BPE, PAR, TPI, AEs
Marmo et al. (46)	Inpatient and outpatient	217	218	59.2 ± 14.8	58.2 ± 15.9	Inverted OBPS	2L PEG+Asc with split dosage	4L PEG with split dosage	BPE, PAR, TPI, AEs
Marmo et al. (46)	Inpatient and outpatient	218	215	57.5 ± 13.8	57.9 ± 14.8	Inverted OBPS	2L PEG+Asc with single dosage	4L PEG with single dosage	BPE, PAR, TPI, AEs
Jansen et al. (23)	Outpatient	102	91	56.6 ± 15.3	59.3 ± 14.1	Non-validated 3-point scale	2L PEG+Asc with split dosage for morning colonoscopy and single dose for afternoon colonoscopy	4L PEG with split dosage for morning colonoscopy and single dose for afternoon colonoscopy	PAR, TPI, AEs
Valiante et al. (13)	Outpatient	166	166	63 (36–82)	65 (42–85)	Aronchick scale score	2L PEG+Asc with split dosage	4L PEG with split dosage	BPE, PAR, Acceptability, AEs
Ponchon et al. (48)	Outpatient	202	198	55.1 ± 12.5	55.9 ± 12.2	Harefield cleaning scale	2L PEG+Asc with split dosage	4L PEG with split dosage	BPE, PAR, Acceptability, WRSP, AEs
Moon et al. (21)	Outpatient	163	164	52.3 ± 11.8	54.0 ± 11.6	Non-validated 5-point scale	2L PEG+Asc with split dosage	4L PEG with split dosage	BPE, PAR, Acceptability, AEs
Rivas et al. (49)	Outpatient	102	104	57.4 ± 7.9	55.9 ± 7.6	OBPS	2L PEG+Asc with single dosage for afternoon colonoscopy	4L PEG with single dosage for afternoon colonoscopy	BPE, PAR, Acceptability, TPI, WRSP, AEs
Kim et al. (44)	Outpatient	159	160	48.0 ± 8.8	45.0 ± 10.7	OBPS	2L PEG+Asc with split dosage	4L PEG with split dosage	BPE, PAR, Acceptability, WRSP, AEs
Jung et al. (43)	Outpatient	63	67	71.3 ± 5.0	71.2 ± 4.4	BBPS	2L PEG+Asc with split dosage	4L PEG with split dosage	BPE, PAR, Acceptability, TPI, WRSP, AEs
Lee et al. (45)	Outpatient	112	114	56 ± 10	55 ± 12	BBPS	2L PEG+Asc with split dosage	4L PEG with split dosage	BPE, Acceptability, TPI, WRSP, AEs

E, experiment group; C, control group; n.r., not reported; OBPS, the Ottawa Bowel Preparation Score; BBPS, Boston bowel preparation scale; BPE, bowel preparation efficacy; WRSP, willingness to repeat the same preparation; TPI, taste of purgative ingested; PAR, patient adherence with regime; Aes, adverse events.

* *proceeding abstract*.

### Methodological Quality of Studies

Considering that three conference articles (47, 50, 51) only provided a simple abstract which essential information cannot be achieved, so we abandoned their assessment of risk of bias. In remaining 10 trials (13, 21–23, 43–46, 48, 49), all of them meticulously described random sequence generation. And seven articles (13, 21, 22, 43, 45, 46, 48) provided a detailed description of conducting allocation concealment. It was extremely difficult to imagine how blinding of patients could be applicable, because participants would know which bowel preparation solutions were ingested. Whereas, eight trials (13, 21, 22, 43, 45, 46, 48, 49) mentioned how to blind endoscopists who implemented this procedures. Furthermore, blinding of outcome assessment have been completed in all trials. In order to reduce attrition bias, five studies (13, 22, 23, 46, 48) adopted ITT analysis to deal with data, seven studies (13, 21, 22, 43, 46, 48, 49) reported the similar dropout rate between two groups and explained the specific reasons that are irrelevant to the study itself. All studies reported outcomes adequately, among them, half of the eligible RCTs (21, 22, 43, 44, 48) were registered in the Clinical Trials.gov., and the remaining articles that cannot provide the protocol identified this conclusion according to methods and participants section. Three trials (22, 45, 48) were funded by the pharmaceutical industry, which may introduce certain potential source of bias. We displayed the risk of bias summary for studies in Figure S1.

### Bowel Preparation Efficacy

**Figure 2:**
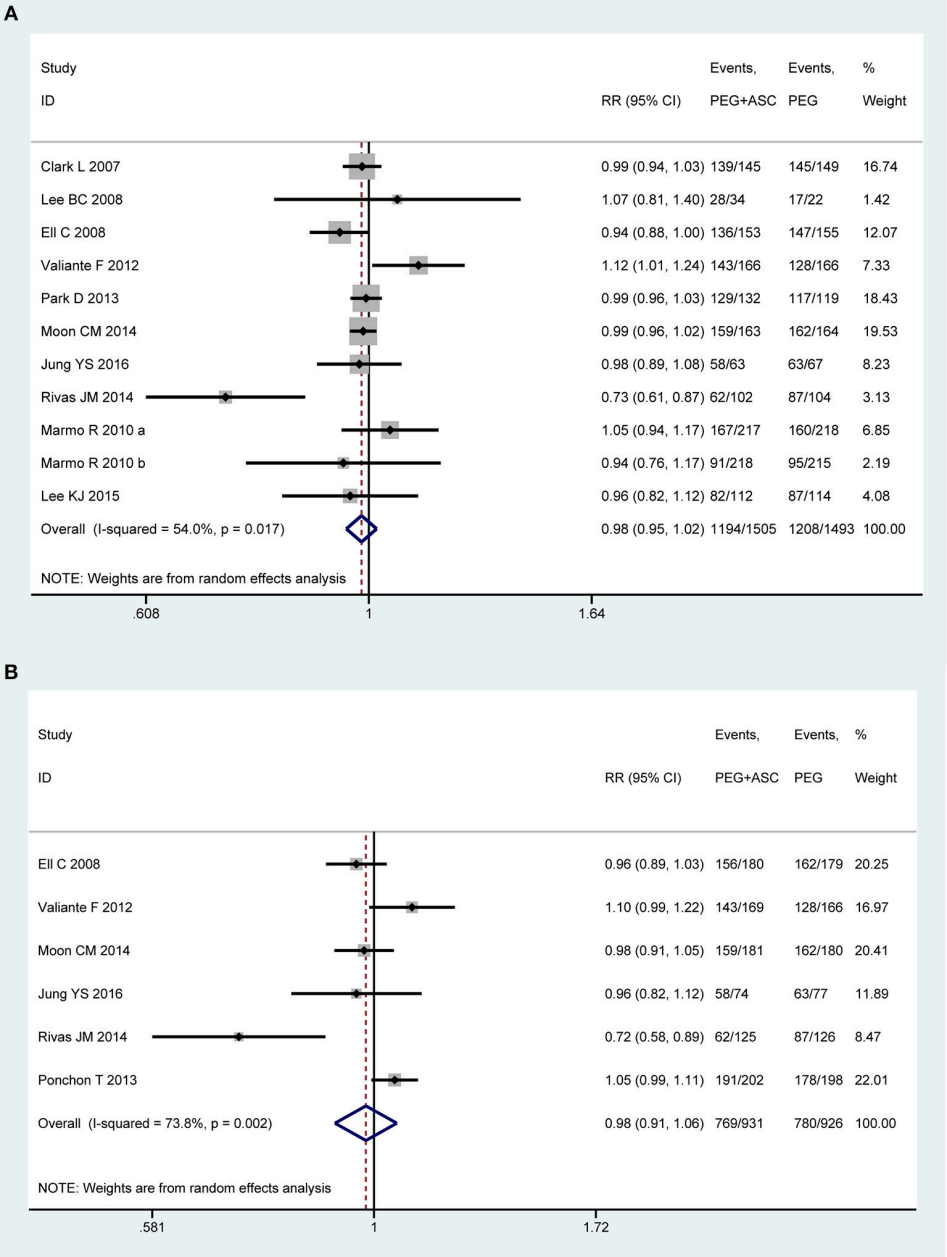
Meta-analysis on bowel preparation efficacy based on PP data **(A)** and ITT data **(B)**. The summary effect estimate (risk ratio, RR) for individual randomized controlled trials (RCTs) are indicated by gray rectangles (the size of the rectangle is proportional to the study weight), with the black horizontal lines representing 95% confidence intervals (CIs). The overall summary effect estimate (risk ratio) and 95% confidence interval are indicated by the blue diamond below. Meta-analysis indicated no difference between 2L PEG plus Asc volume and 4L PEG regimes in terms of bowel preparation efficacy.

Eleven trials (13, 21, 22, 43, 45–47, 49–51) involving 2,998 participants investigated bowel preparation efficacy in PP analysis. Statistical heterogeneity was detected across the included studies [*P* = 0.017, *I*^2^ = 54%], and then a random-effect model was adopted to calculate estimate. Pooled result suggested that the efficacy of 2L PEG plus Asc is not inferior to the 4L PEG regimen for bowel cleansing [RR = 0.98, 95% CI is 0.95 to 1.02, *P* = 0.34] (Figure 2A). Six RCTs (13, 21, 22, 43, 48, 49) involving 1,857 participants reported this outcome variable in ITT analysis, which included 931 and 926 patients between groups, respectively. Substantial heterogeneity in basic clinical characteristics and methodology of eligible studies was considered [*P* = 0.002, *I*^2^ = 73.8%], and a random-effect model was conducted to summarize effect size. Pooled result was similar to the one in PP analysis which well-suggested that the summary effect size was robust [RR = 0.98, 95% CI is 0.91 to 1.06, *P* = 0.61] (Figure 2B).

In order to test the heterogeneity and robust behavior in terms of bowel preparation efficacy, we also performed the sensitivity analyses according to approach of drinking solution and the type of patients. The sensitivity analysis based on outpatients was robust in terms of PP analysis [*P* = 0.001, *I*^2^ = 78.7%, RR = 0.971, 95% CI is 0.926 to 1.017, *P* = 0.213] and ITT analysis [*P* = 0.001, *I*^2^ = 77.5%, RR = 0.981, 95% CI is 0.891 to 1.079, *P* = 0.692]. Moreover, sensitivity analysis based on split-dose was also robust [PP analysis: RR = 1.005, 95% CI is 0.976 to 1.035, *P* = 0.731; ITT analysis: RR = 1.012, 95% CI is 0.975 to 1.051, *P* = 0.523] although heterogeneity for PP analysis [*P* = 0.140, *I*^2^ = 34.7%] and ITT analysis [*P* = 0.109, *I*^2^ = 47%] were all reduced.

We also undertook TSA on these data in PP analysis. The number of patients included in the meta-analysis did not exceed the RIS, but crossed below the futility boundaries. Therefore, within the set assumptions for confidence and effect size, we are therefore able to infer neither 2L PEG combined with Asc group nor 4L PEG group is more than 5% more effective than the other, and extra resources should not be wasted to plan further studies (Figure S2A).

### Compliance With the Regimen

**Figure 3:**
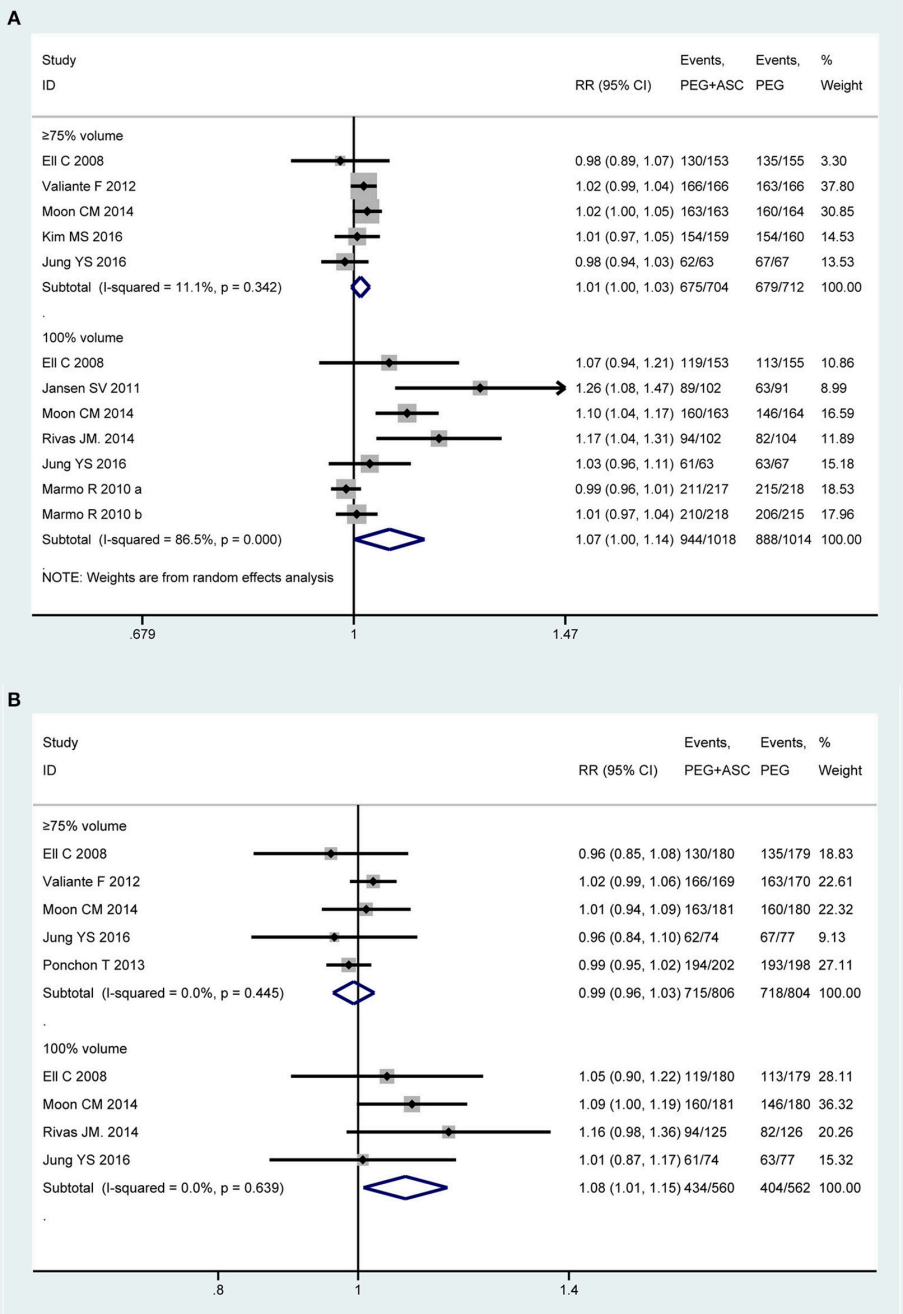
Meta-analysis on compliance to the regimen based on PP data **(A)** and ITT data **(B)**. The summary effect estimate (risk ratio, RR) for individual randomized controlled trials (RCTs) are indicated by gray rectangles (the size of the rectangle is proportional to the study weight), with the black horizontal lines representing 95% confidence intervals (CIs). The overall summary effect estimate (risk ratio) and 95% confidence interval are indicated by the blue diamond below. Meta-analysis indicated a better compliance with recommend regime in 2L PEG plus ASC group when the full amounts of solution were ingested.

Nine trials (13, 21–23, 43, 44, 46, 49, 50) involving 2,739 participants investigated the compliance to the regimen in PP analysis. Among them, one study (50) only provided an abstract, and the concept of compliance can't be defined clearly, eventually eight trials made the quantitative analysis. We divided the eligible studies into two subgroups based on consumption of cleansing solution that participants have ingested: (1) consumption of ≥75% but < 100% of total amount recommended (≥75% intake group) and (2) consumption of 100% of total amount recommended (100% intake group). Homogeneity existed among seven RCTs that fell into ≥75% intake group [*P* = 0.34, *I*^2^ = 11.1%], but statistical heterogeneity is present among five studies that fell into 100% intake group [*P* < 0.001, *I*^2^ = 86.5%]. Therefore, a random-effects model was used. Pooled result showed that a better tendency was seen for the 2L PEG plus Asc group when patients ingested the entire solution as prescribed, while no differences in the rate of ≥75% consumption of the preparation was detected [RR = 1.01, 95% CI is 1.00 to 1.03, *P* = 0.143 for ≥75% intake group and RR = 1.068, 95% CI is 1.001 to 1.138, *P* = 0.046 for 100% intake group] (Figure 3A). Six trials (13, 21, 22, 43, 48, 49) reported the compliance to the regimen in ITT analysis, which included 931 and 930 patients between groups, respectively. No heterogeneity in basic clinical characteristics and methodology of eligible studies was found [*P* = 0.45, *I*^2^ = 0% for ≥75% intake group and *P* = 0.64, *I*^2^ = 0% for 100% intake group], and a fixed-effect model was conducted to summarize effect size. Pooled result was similar to the one in PP analysis which well-suggested that the summary effect size was robust [RR = 1.00, 95% CI is 0.98 to 1.03, *P* = 0.87 for ≥75% intake group and RR = 1.08, 95% CI is 1.01 to 1.15, *P* = 0.02 for 100% intake group] (Figure 3B).

We also undertook TSA on these data in PP analysis. In ≥75% intake group, the cumulative Z-curve didn't reach the RIS, but crossed the futility boundaries, in which case, it would be inferred that the experimental intervention is not superior to the control intervention, and extra resources should not be wasted to plan further studies (Figure S2B). In 100% intake group, the cumulative *Z*-curve didn't exceed the RIS, but crossed the O'Brien-Fleming boundaries, in which case, it would be inferred that the experimental intervention is superior to the control intervention, and extra resources should not be wasted to plan further studies (Figure S2C).

### Willingness to Retake the Same Regime

**Figure 4:**
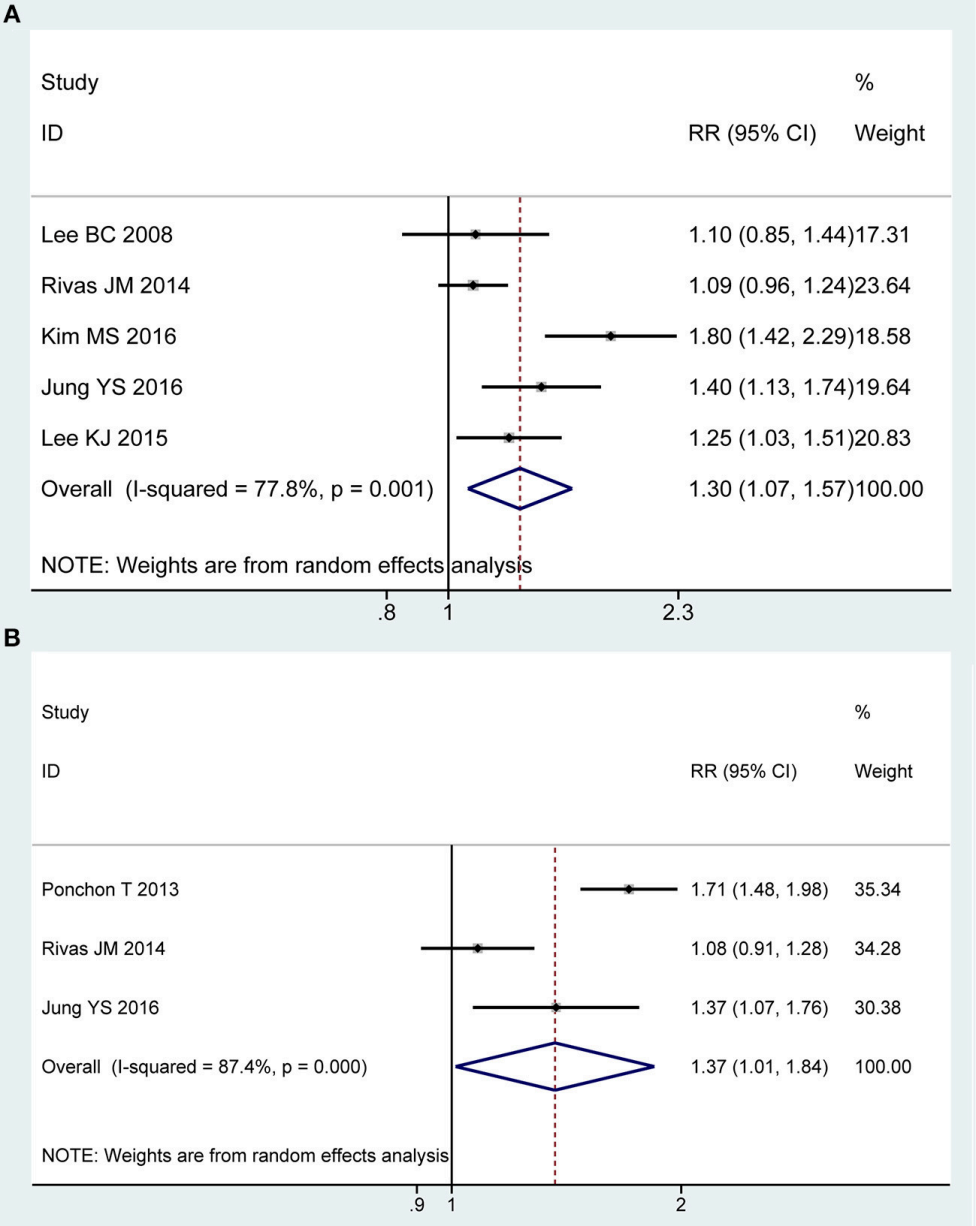
Meta-analysis on willingness to retake the same regime based on PP data **(A)** and ITT data **(B)**. The summary effect estimate (risk ratio, RR) for individual randomized controlled trials (RCTs) are indicated by gray rectangles (the size of the rectangle is proportional to the study weight), with the black horizontal lines representing 95% confidence intervals (CIs). The overall summary effect estimate (risk ratio) and 95% confidence interval are indicated by the blue diamond below. Meta-analysis indicated a better preference to repeat the same regime when 2L PEG plus Asc vs. 4L PEG regimes.

Five of all trials (43–45, 49, 50) involving 937 participants investigated the willingness to retake the same regime in PP analysis. Statistical heterogeneity was detected across the included studies [*P* = 0.001, *I*^2^ = 77.8%], and then a random-effect model was adopted to summarize mean effect size. Pooled result suggested that the 2L PEG plus Asc group was more likely to be willing to repeat the preparation with the same solution than the 4L PEG group [RR = 1.30, 95% CI is 1.07 to 1.57, *P* = 0.007] (Figure 4A). Three RCTs (43, 48, 49) reported the willingness to retake the same regime in ITT analysis, which included similar number of patients between groups, respectively. Substantial heterogeneity in basic clinical characteristics and methodology of eligible studies was considered [*P* < 0.001, *I*^2^ = 87.4%], and a random-effect model was conducted to summarize effect size. Pooled result was similar to the one in PP analysis which well-suggested that the summary effect size was robust [RR = 1.37, 95% CI is 1.01 to 1.84, *P* = 0.04] (Figure 4B).

We also undertook TSA on these data in PP analysis, the cumulative Z-curve for included trials reached the RIS, and crossed the O'Brien-Fleming boundaries, in which case, it would be inferred that willingness to repeat the same regime was higher with 2L PEG plus Asc group than with 4L PEG group, and future similar studies are futile (Figure S2D).

### Acceptability to Regime

**Figure 5:**
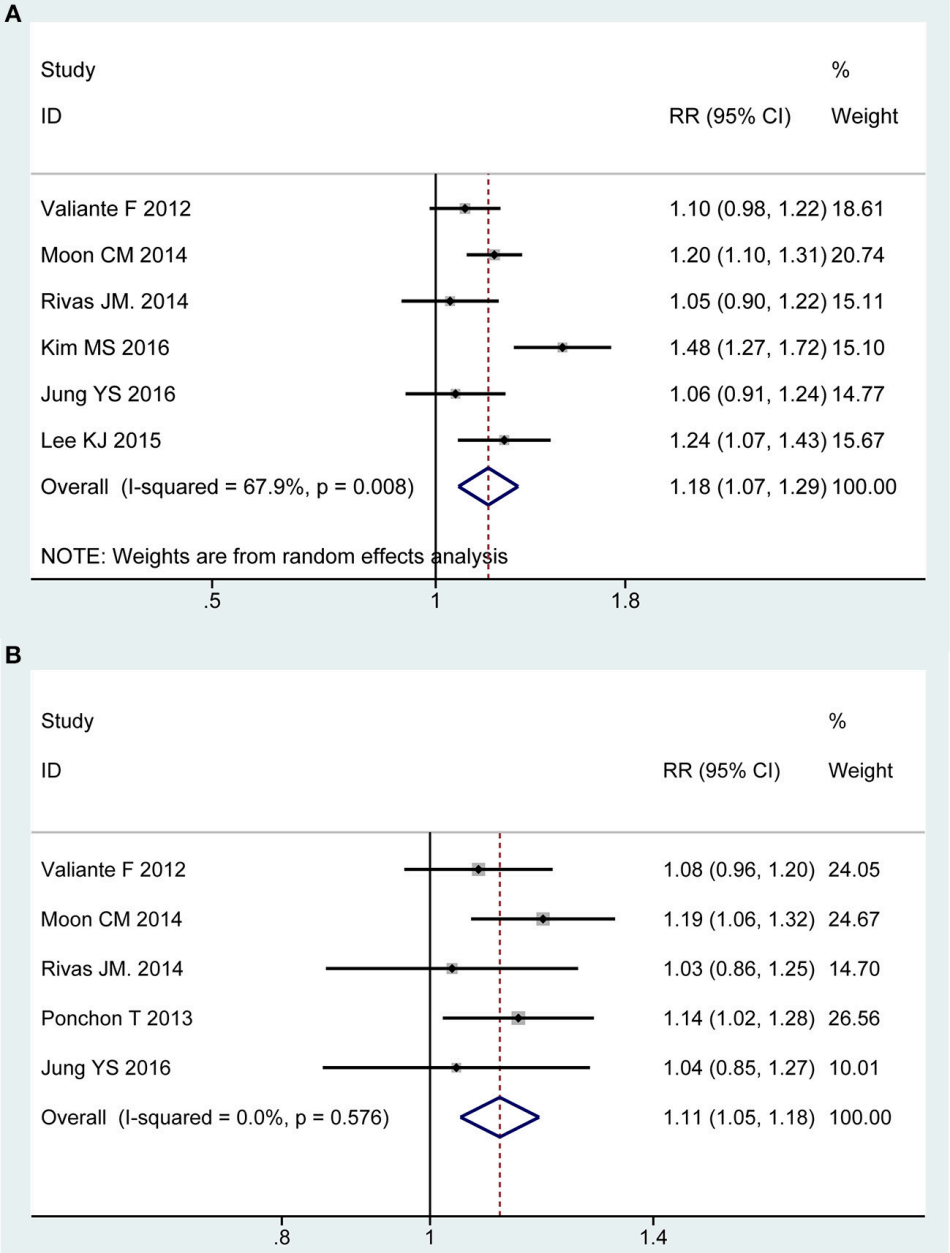
Meta-analysis on acceptability to regime based on PP data **(A)** and ITT data **(B)**. The summary effect estimate (risk ratio, RR) for individual randomized controlled trials (RCTs) are indicated by gray rectangles (the size of the rectangle is proportional to the study weight), with the black horizontal lines representing 95% confidence intervals (CIs). The overall summary effect estimate (risk ratio) and 95% confidence interval are indicated by the blue diamond below. Meta-analysis indicated patient acceptability was higher for 2L PEG plus Asc regime than for 4L PEG regime.

Six of all trials (13, 21, 43–45, 49) involving 1,540 participants investigated the acceptability to regime in PP analysis. Statistical heterogeneity was detected across the included studies [*P* = 0.008, *I*^2^ = 67.98%], and then a random-effect model was adopted to summarize mean effect size. Pooled result suggested that patient acceptability was higher for 2L PEG plus Asc group than for control group [RR = 1.18, 95% CI is 1.07 to 1.29, *P* = 0.001] (Figure 5A). Five RCTs (13, 21, 43, 48, 49) reported the acceptability to regime in ITT analysis, which included similar number of patients between groups, respectively. No heterogeneity in basic clinical characteristics and methodology of eligible studies was considered [*P* = 0.58, *I*^2^ = 0%], and a fixed-effect model was conducted to summarize effect size. Pooled result was similar to the one in PP analysis which well-suggested that the summary effect size was robust [RR = 1.12, 95% CI is 1.05 to 1.18, *P* < 0.001] (Figure 5B).

We also undertook TSA on these data in PP analysis, the cumulative Z-curve for included trials reached the RIS, and crossed the O'Brien-Fleming boundaries. Therefore, we are able to infer that acceptability to regime was higher with 2L PEG plus ASC group than with 4L PEG group, and additional studies with large sample sizes should not be required in the future (Figure S2E).

### Safety

**Figure 6:**
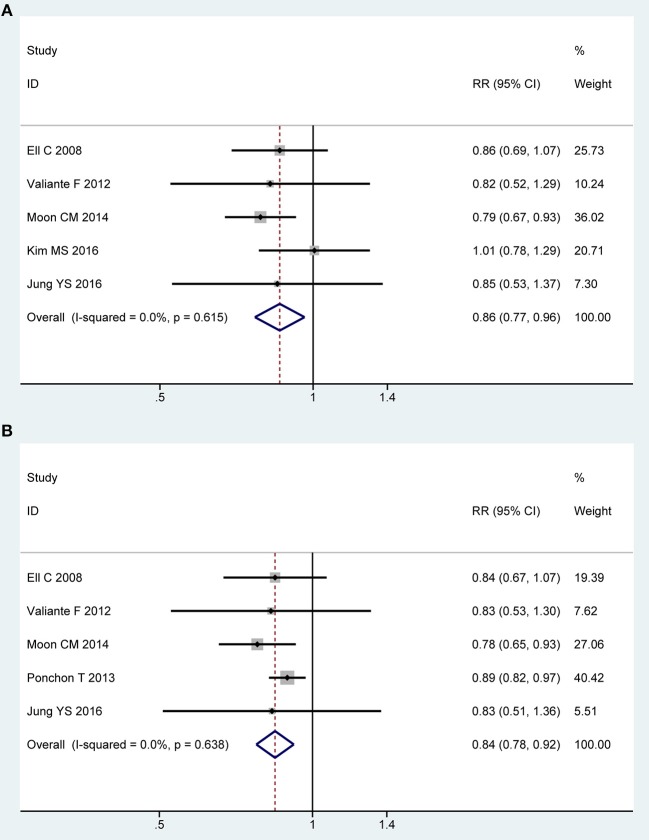
Meta-analysis on overall AEs based on PP data **(A)** and ITT data **(B)**. The summary effect estimate (risk ratio, RR) for individual randomized controlled trials (RCTs) are indicated by gray rectangles (the size of the rectangle is proportional to the study weight), with the black horizontal lines representing 95% confidence intervals (CIs). The overall summary effect estimate (risk ratio) and 95% confidence interval are indicated by the blue diamond below. Meta-analysis indicated a significant difference when 2L PEG plus Asc vs. 4L PEG regime in terms of overall adverse events.

Five of all trials (13, 21, 22, 43, 44) involving 1,416 participants investigated overall adverse events in PP analysis. No heterogeneity was detected across the included studies [*P* = 0.62, *I*^2^ = 0%], and then a fixed-effect model was adopted to summarize mean effect size. Pooled result suggested that overall adverse events was lower for 2L PEG plus Asc group than for control group [RR = 0.86, 95% CI is 0.77 to 0.96, *P* = 0.009] (Figure 6A). Five RCTs (13, 21, 22, 43, 44) reported the overall adverse events in ITT analysis, which included 806 and 804 patients between groups, respectively. No heterogeneity in basic clinical characteristics and methodology of eligible studies was considered [*P* = 0.64, *I*^2^ = 0%], and a fixed-effect model was conducted to summarize effect size. Pooled result was similar to the one in PP analysis which well-suggested that the summary effect size was robust [RR = 0.84, 95% CI is 0.77 to 0.92, *P* < 0.001] (Figure 6B). Treatment-related adverse effects mainly including abdominal cramps (*n* = 1,496), nausea (*n* = 1,529), and vomiting (*n* = 1,529) were analyzed. Pooled results are summarized in electroc Supplemetary Table 1.

We only undertook TSA on these data from overall adverse events in PP analysis. For other special adverse effects, due to too little information use, boundary required sample size is ignored. Though the cumulative *Z*-curve for included trials didn't reach the RIS, it crossed the O'Brien-Fleming boundaries. Therefore, we are therefore able to infer that low-volume 2L PEG plus Asc showed significantly fewer overall adverse events than did 4L PEG, and extra resources should not be wasted to plan further studies (Figure S2F).

### Taste of Purgative Ingested

Seven studies (22, 23, 43, 45, 46, 49, 50) reported taste of purgative ingested as an outcome. Because different non-validated scoring systems have been applied for assessing this factor in different studies, which causes difficulties in defining the cutoff of good or bad taste and analyzing the results, a descriptive analysis was performed. Five trials (22, 23, 45, 46, 50) found that participants who randomized to the 2L PEG plus Asc group rated the taste better than those who received 4L PEG solution. Conversely, in the remaining articles (43, 49), although the difference was not statistically significant, however, a trend was noted for a more pleasant taste in the 4L PEG as compared to 2L PEG plus Asc.

### Publication Bias

For primary outcomes (bowel preparation efficacy), we performed a funnel plot, which often be depicted to identify the existence of publication bias. The funnel plot displayed symmetry, thereby indicating that no small study bias possibly exists. Meanwhile, the Egger's test result also revealed similar result (*P* = 0.383) (Figure S3). Other indicators including few studies didn't meet the conditions.

## Discussion

Subjects' participation and adequate bowel cleansing are the essential requirements for a high-quality colonoscopy (1, 25, 52). Therefore, the ideal colon cleansing should be capable of evacuating the colon from all fecal material without damaging its mucosa, causing no discomfort, and minimizing fluids and electrolyte disturbances (53, 54). Traditional 4L PEG regime has been used worldwide for its high efficacy, lower price and superior safety (55, 56). But volume-related discomfort and unpleasant taste may deter the acceptability with colonoscopy, which is closely related to subject's attendance (57). Poor acceptability will impair the willingness to take the examination in the future (14, 15). Recently, low volume PEG regime shows a better toleration under the condition that its cleanliness is equal to that of traditional 4L PEG regimen (16, 21, 22, 48). Although a previous meta-analysis has confirmed low volume PEG plus Asc is an effective alternative, several limitations harmed the reliability of pooled results. Moreover, some potential RCTs have been published recently. Thus, it is essential to further determine the comparative role between low volume PEG regime and traditional large volume one in bowel preparation before colonoscopy.

### Summary of Main Results

This meta-analysis found that 2L PEG plus Asc is as effective as 4L PEG solution with respect to successful bowel cleansing in non-selected population. Meanwhile, it can enhance patient willingness to repeat the same regime, acceptability, and compliance when 100% of the prescribed solution was ingested, and can decrease the overall AEs. However, whether Asc may improve palatability of PEG solution will require additional studies with large-scale to establish. Furthermore, the results of TSA confirmed the efficacy of 2L PEG plus Asc on bowel preparation efficacy, compliance, willingness, and acceptability to regime and overall AEs.

While we failed to find a difference between the two groups when patients drank ≥75% of the gut cleaning solution, significantly more patients in the 4L PEG group reflected having difficulty consuming the total amount of bowel preparation solution. There are some explanations to interpret why this discrepancy occurs: (a) despite 2L PEG plus Asc has better acceptability, researchers can partly improve the compliance through continuous education and the delivery of preparation instructions (2, 58, 59). These means addressed patient knowledge and belief barriers to quality colonoscopy preparation; (b) our subjects were patients who were more concerned about their health than the normal population. When health-care professionals counseled patients to complete intake of the solution to ensure a safe and effective procedure, they would be better able to understand the importance of compliance; and (c) several studies (22, 46) recruited hospitalized patients who may achieve higher compliance. Nonetheless, it remains obvious that a considerable proportion of patients is unable or unwilling to drink a large volume of unpalatable fluid.

Besides, it is generally believed that the addition of Asc can mask the unpleasant taste of PEG preparation (13, 22, 48), resulting in improved acceptability and patient compliance. However, contrary to our expectations, the present study failed to get it. Maybe we can speculate the palatability didn't contribute significantly to patient compliance and acceptability to regimen, in other words, there was no relationship between the taste of the cleansing agent and the probability of a positive response.

### Limitations of the Present Study

There are several limitations that need to be acknowledged in our study. Firstly, there is little uniformity concerning bowel cleansing evaluation, with endoscopists using several scale tools, and criteria to definite the cutoff of adequate colon cleansing. However, all these appraisal tools place emphasis on similar aspects, including the removable volume of clear liquid and (or) fecal residue and the impact of the surplus on mucosal visibility. This point greatly reduces the incidence rate of scoring bias. Secondly, it was hard to conceive how blinding of patients could be applicable. Assessment of subjective outcomes in this article (willingness to retake the same regime, acceptability to regime, taste of purgative ingested) mainly depended on the personal judgment of participants, it is unknown if this may have introduced bias on the evaluation of these variables. Thirdly, though there was heterogeneity among comparisons regarding variations in dosing regimens (e.g., non-split or split schedule; morning or afternoon colonoscopy), in dietary restrictions, and in demographics and population types. Due to the randomization, there is a high probability that they would have been evenly distributed between the intervention arms, hence a confounding effect on the final results could be excluded. Finally, Gimeno-Garcia AZ et al found that 4L PEG based preparation is superior to 2L PEG Asc based preparation in patients with past history of poor bowel preparation (60), and thus it must be emphasized that 2L PEG plus Asc is as effective as taking 4L PEG solution in non-selected population because these results may be not generalized to hard to prepare group.

### Implications for Practice and Further Research

In our review, less than half of primary studies (13, 21, 22, 48, 49) scheduled all subjects for examination in concentrated time to reduce bias related to procedure time, but didn't determine the actual time at which the preparation was finalized. Siddiqui et al. (5) reported that for every additional hour that the patient waits between the last dose of bowel preparation agents and the colonoscopy start time, the chance to achieve an inadequate cleansing in the right colon increases by up to 10%. Several previous studies have demonstrated that the sooner the procedure is conducted from ingestion, the higher the chance of achieving a clean bowel (46, 61–63), and European Society of Gastrointestinal Endoscopy further recommends (55) that the delay between the last fluid intake and colonoscopy should be no longer than 4 h (strong recommendation, moderate quality evidence). So in the design stage of study, researchers should take this factor into account to provide a more standardized, scientific, rationalized way for clinical use.

## Conclusions

According to our data, 2L PEG plus Asc appears to be a more patient-friendly preparation considering both efficacy and tolerability. This 2L PEG plus Asc regimen is at least as effective as 4L PEG solution, with clear advantages in terms of patient willingness, acceptability, compliance (100% intake) and safety. However, there is no consensus regarding the effect of the taste of the cleansing agent for colonoscopy.

## Author Contributions

L-JY, XT, Y-PP, and W-QC were involved with study conception and design. L-JY and X-LL were involved in data acquisition. L-JY and BS performed statistical analysis. XT and HC interpreted the data and results of the analyses. L-JY and XT drafted the manuscript, which was critically revised for intellectual content by XT and W-QC. All authors read and approved the final manuscript.

### Conflict of Interest Statement

The authors declare that the research was conducted in the absence of any commercial or financial relationships that could be construed as a potential conflict of interest.
