# Supplementary material for: Low-Volume Polyethylene Glycol Improved Patient Attendance in Bowel Preparation Before Colonoscopy: A Meta-Analysis With Trial Sequential Analysis
Source: Front Med (Lausanne). 2019 May 6;6:92. doi: 10.3389/fmed.2019.00092 (PMC6512395; doi:10.3389/fmed.2019.00092)
Supplement: Supplementary file 1 [file Data_Sheet_1.PDF]

	Q1	Q2	Q3	Q4	Q5	Q6	Q7
Ell C 2008	L	L	L	L	L	L	H
Marmo R 2010	L	L	L	L	L	L	L
Jansen SV 2011	L	U	U	L	L	L	L
Valiante F 2012	L	L	L	L	L	L	L
Ponchon T 2013	L	L	L	L	L	L	H
Moon CM 2014	L	L	L	L	L	L	L
Rivas JM 2014	L	U	L	L	L	L	L
Kim MS 2016	L	U	U	L	L	L	L
Jung YS 2016	L	L	L	L	L	L	L
Lee KJ 2015	L	L	L	L	U	L	H

Figure.S1 Risk of bias summary. Q1, random sequence; Q2, allocation concealment; Q3, blinding of participants and personnel; Q4, blinding of outcome assessment; Q5, incomplete outcome data; Q6, selective reporting; Q7, other bias; h, high risk of bias; l, low risk of bias; u, unclear risk of bias.

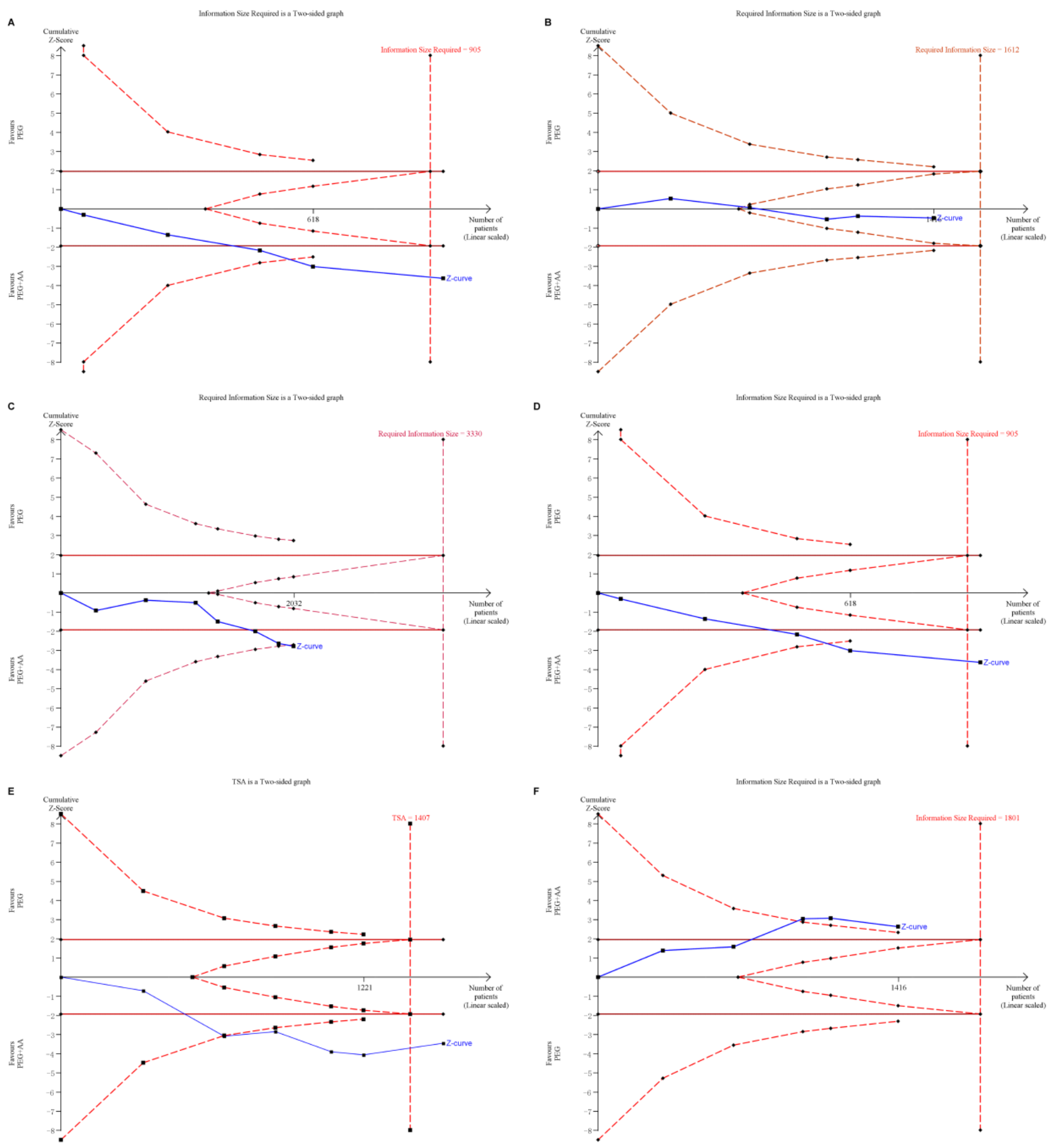

Figure. S2 Trial sequential analysis on all outcomes. The red dashed lines represent the trial sequential monitoring boundaries and the futility boundarie, and the solid blue line is the cumulative Z-curve. **(A) bowel preparation efficacy based on PP data.** The required information size (RIS) to demonstrate or reject a 5% relative increase in benefit on efficacy of colon preparation with a control group proportion of 83%, an alpha of 5% and a beta of 20% is 4007 patients (vertical red line). Although the cumulative Z-curve did not accrued the RIS, but crossed below the futility boundaries. The trial sequential analysis confirmed the evidence which no difference in efficacy of gut cleansing was observed between the two groups. **(B) compliance to the regimen based on PP data (≥ 75% volume).** The required information size to demonstrate or reject a 5% relative increase in benefit on patient compliance to the regimen with a control group proportion of 90%, an alpha of 5% and a beta of 20% is 1612 patients (vertical red line). The cumulative Z-curve didn’t reach the RIS, but crossed below the the futility boundaries. The trial sequential analysis confirmed the evidence which no significant difference was evident between the two groups with regard to compliance to the regimen. **(C) compliance to the regimen based on PP data (100% volume).** The required information size to demonstrate or reject a 5% relative

increase in benefit on compliance to the regimen with a control group proportion of 80% (the average control group event proportion), an alpha of 5% and a beta of 20% is 3330 patients (vertical red line). The cumulative Z-curve didn't reach the RIS, but crossed the O'Brien-Fleming boundaries. The trial sequential analysis confirmed the evidence which low volume PEG regime was superior to standard volume PEG regime for patient compliance to the regimen. **(D) willingness to retake the same regime based on PP data.** The required information size to demonstrate or reject a 20% relative increase in benefit on willingness to retake the same regime with a control group proportion of 63% (the average control group event proportion), an alpha of 5% and a beta of 20% is 905 patients (vertical red line). The cumulative Z-curve not only surpassed the RIS, but also crossed the O'Brien-Fleming boundaries. The trial sequential analysis confirmed the evidence that patients receiving low volume solution expressed more willingness to repeat the same preparation than high volume one. **(E) acceptability to regime based on PP data.** The required information size to demonstrate or reject a 15% relative increase in benefit on acceptability to regime with a control group proportion of 74%(the average control group event proportion), an alpha of 5% and a beta of 20% is 1407 patients (vertical red line). The cumulative Z-curve not only surpassed the RIS, but also crossed the O'Brien-Fleming boundaries. The trial sequential analysis confirmed the evidence that PEG+ASC solution resulted in improved patient acceptability. **(F) overall AEs based on PP data.** The required information size to demonstrate or reject a 20% relative decrease in benefit on overall adverse events with a control group proportion of 29%(the average control group event proportion), an alpha of 5% and a beta of 20% is 1801 patients (vertical red line). The cumulative Z-curve didn't reach the RIS, but crossed the O'Brien-Fleming boundaries. The trial sequential analysis confirmed the evidence that there was no significant difference in over adverse events between the two groups.

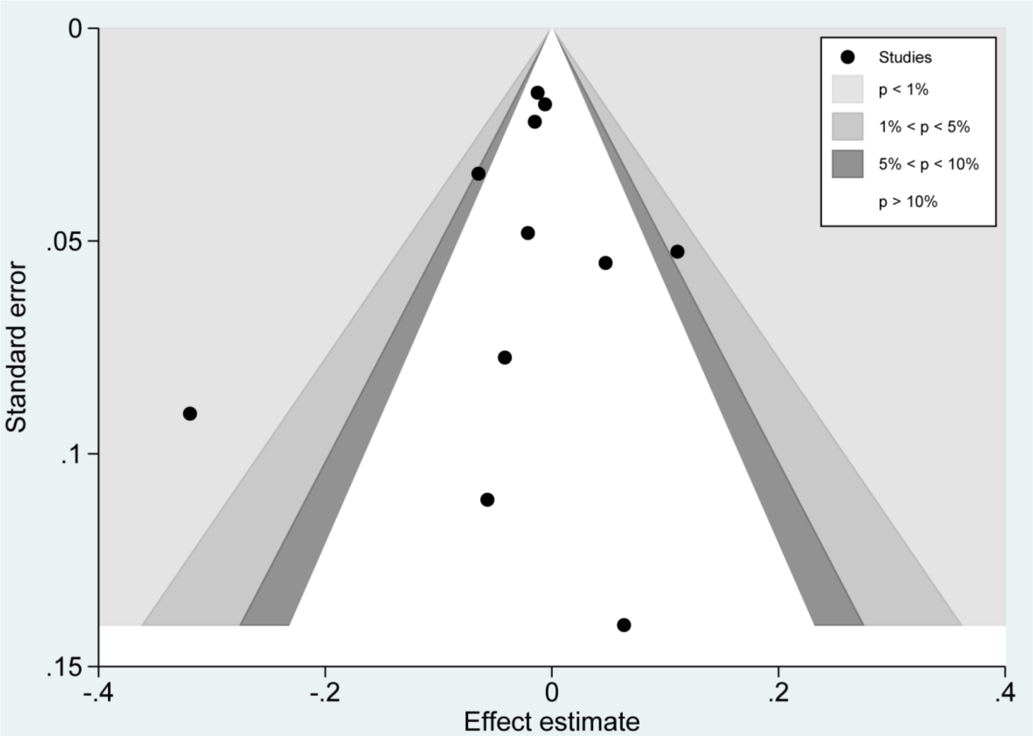

Figure. S3 Funnel plot of bowel preparation efficacy. The vertical axis represented the standard error (SE) of effect size and x axis indicated the ratio between effect size and pooled effect. Symmetrical funnel plot indicated no small study effect.

**Electroc supplemetary table 1. Outcomes of AEs analyzed between 2L PEG plus ASC and 4L PEG before colonoscopy**						
AEs	Studies number	RR	95%CL	I^2	P-value	Analysis type
abdominal pain	7	0.85	0.67-1.08	0.0%	0.19	PP
	5	0.88	0.61-1.25	0.0%	0.47	ITT
nausea	6	0.82	0.68-0.99	0.0%	0.03	PP
	5	0.78	0.64-0.96	0.0%	0.02	ITT
vomiting	6	0.61	0.43-0.87	0.0%	0.01	PP
	5	0.61	0.41-0.90	0.0%	0.01	ITT
